# The fusiform gyrus exhibits differential gene-gene co-expression in Alzheimer's disease

**DOI:** 10.3389/fnagi.2023.1138336

**Published:** 2023-05-15

**Authors:** Arthur Ribeiro-dos-Santos, Leonardo Miranda de Brito, Gilderlanio Santana de Araújo

**Affiliations:** ^1^Programa de Pós-graduação em Genética e Biologia Molecular, Laboratório de Genética Humana e Médica, Instituto de Ciências Biológicas, Universidade Federal do Pará, Belém, Brazil; ^2^Centro de Informática, Universidade Federal de Pernambuco, Recife, Brazil

**Keywords:** co-expression networks, differential co-expression networks, hub genes, regulatory networks, fusiform gyrus, Alzheimer's disease, brain tissue

## Abstract

Alzheimer's Disease (AD) is an irreversible neurodegenerative disease clinically characterized by the presence of β-amyloid plaques and tau deposits in various regions of the brain. However, the underlying factors that contribute to the development of AD remain unclear. Recently, the fusiform gyrus has been identified as a critical brain region associated with mild cognitive impairment, which may increase the risk of AD development. In our study, we performed gene co-expression and differential co-expression network analyses, as well as gene-expression-based prediction, using RNA-seq transcriptome data from post-mortem fusiform gyrus tissue samples collected from both cognitively healthy individuals and those with AD. We accessed differential co-expression networks in large cohorts such as ROSMAP, MSBB, and Mayo, and conducted over-representation analyses of gene pathways and gene ontology. Our results comprise four exclusive gene hubs in co-expression modules of Alzheimer's Disease, including *FNDC3A, MED23, NRIP1*, and *PKN2*. Further, we identified three genes with differential co-expressed links, namely *FAM153B, CYP2C8*, and *CKMT1B*. The differential co-expressed network showed moderate predictive performance for AD, with an area under the curve ranging from 0.71 to 0.76 (+/− 0.07). The over-representation analysis identified enrichment for Toll-Like Receptors Cascades and signaling pathways, such as G protein events, *PIP2* hydrolysis and *EPH-Epherin* mechanism, in the fusiform gyrus. In conclusion, our findings shed new light on the molecular pathophysiology of AD by identifying new genes and biological pathways involved, emphasizing the crucial role of gene regulatory networks in the fusiform gyrus.

## 1. Introduction

Alzheimer's Disease (AD) is an irreversible neurodegenerative disease that leads to severe dementia and incremental disability in adults, largely in the elderly (Alzheimer's Association, 2022). Basic research has expanded our understanding of the multifaceted pathophysiological mechanisms of Alzheimer's Disease (AD), affirming that many previous molecular changes arise earlier before its severe form (Aisen et al., 2017).

Currently, molecular factors of AD rely on β-amyloid plaques and neurofibrillary tau proliferation in the neocortex (Jack Jr et al., 2018). Along with it, several molecular layers involved in AD make our systematic understanding insufficient concerning the complexity of the disease, such as gene and miRNA regulation (Souza et al., 2016; Brito et al., 2020), mitochondrial genetics (Cavalcante et al., 2022; Song et al., 2022), and especially gene-gene interactions in AD across brain regions (Lancour et al., 2020; Wang et al., 2021). Although there are no well-defined points of molecular distinction, clinical and genetic studies divide AD into early-onset AD associated with molecular modifications of *APP, PSEN1* and *PSEN2* (Wan et al., 2020) and the late-onset AD that is mainly associated with risk variants in the *APOE* gene, while recently, 75 genes are reported as risk factors in multi-ethnic studies of AD (Bellenguez et al., 2022).

Recently, the fusiform gyrus, which is a brain region that plays roles in the vision for perception, object recognition, and reading has gained attention in epigenetic studies (Srinivasan et al., 2020). Specific changes in functional connectivity of the fusiform gyrus have been reported in mild cognitive impairment, considered a risk factor of conversion to AD (Ma et al., 2020). Chang et al. (2016) indicated atrophy of the fusiform gyrus as a consequence of amyloid load within the hippocampus. Besides, the mechanisms involved in AD pathology concerning the fusiform gyrus remain underexplored. Ma et al. (2020) considers that AD-linked genes in the fusiform gyrus may be critical in AD onset progression and, therefore, stand promising targets for early diagnosis and therapy. Thus, an investigation into the molecular mechanisms in the fusiform gyrus of AD patients is necessary.

In this study, we utilized co-expression networks and differential co-expression network analysis to gain a better understanding of gene-gene interactions in the AD fusiform gyrus. Our objective was to identify potential genes that could predict AD and contribute to the overall understanding of gene-gene interactions in the fusiform gyrus of AD patients. The results of our analyses revealed a total of seven genes, four of which were identified through the co-expression analysis and three through the differential co-expression analysis. We also developed a differential gene co-expression network with moderate predictive performance, which was combined with extreme Gradient Boosting (XGBoost) to predict AD.

## 2. Materials and methods

### 2.1. RNA-seq from fusiform gyrus of Alzheimer's disease and neurologically normal post-mortem

The RNA-seq transcriptome dataset was obtained from the Gene Expression Omnibus under accession number GSE125583. The RNA was extracted from the fusiform gyrus of post-mortem tissue from individuals with AD or who were neurologically normal as controls (NC) (*n* = 289), with age ranges from 60 to 103 years old (Srinivasan et al., 2020). Samples were extracted from the fusiform gyrus of neurologically normal controls (*n* = 70, aged 71 to 103) and AD patients (*n* = 219, aged 60 to 103), and were sequenced using the Illumina HiSeq 2,500 sequencer in single-end read mode.

The process of downloading the *.sra* files for each sample were carried out using the prefetch tool from *SRA Toolkit* (Leinonen et al., 2010). Subsequently, all *.sra* files were converted to *.fastq* files with the help of *fastq-dump*. Quality control checks were performed on each sample both before and after RNA-seq preprocessing. The Trimmomatic package was utilized to remove Illumina adapters, as well as to trim and filter low-quality reads and bases. Trimmomatic was executed in Single End Mode, using the ILLUMINACLIP command to remove adapters that were specified in the TruSeq3-SE.fa file, with the threshold values for seed mismatches, palindrome clip, and simple clip set to 2, 30, and 10, respectively. Other commands, such as LEADING, TRAILING, SLIDINGWINDOW, and MINLEN, had their values set to 3, 3, 4:15, and 36, respectively. Read alignment was performed using STAR (Dobin et al., 2013), with the hg19 genome reference being employed. STAR was configured to recognize that a read overlaps a gene, regardless of whether it maps to the same or opposite strand. Read counting was carried out with HTseq, and the gene symbols were annotated using the HUGO Gene Nomenclature Committee (Anders et al., 2015).

The process of transcript filtering was carried out in three distinct steps. Firstly, the read counts were normalized using the counts per million. Subsequently, transcripts were filtered based on the global average of reads, where the read count per transcript and per sample were observed. Lastly, transcripts were removed if the sum of their counts across all samples was less than the global average of reads. After following this process, we carried out a transcript and sample filtering according to the best practices for co-expression and differential co-expression analysis. These best practices include using 20 or more samples per group, ensuring both a large volume of reads per sample (around 10 million) and a high read depth, which corresponds to the number of times each nucleotide was read for each sequence (Ballouz et al., 2015).

### 2.2. The RNA-seq harmonization study (ROSMAP, MSBB, and Mayo Cohorts)

In addition to fusiform gyrus RNA-seq data, we accessed RNA-seq data from three large cohorts described as follows:

- ROSMAP stands for Religious Order Study (ROS) and Memory and Aging Project (MAP), which are two longitudinal clinical-pathological cohort studies conducted by RUSH University (Bennett et al., 2018). ROS focuses on memory, motor, and functional problems related to aging and Alzheimer's disease in Catholic orders, while MAP investigates the decline in cognitive and motor function and the risk of Alzheimer's disease in the general population. Clinical data and transcriptomic RNA-seq data from post-mortem donors were accessed from ROSMAP, including tissues such as the dorsolateral prefrontal cortex (AD = 308, NC = 148), frontal cortex (AD = 24, NC = 25), head of the caudate nucleus (AD = 178, NC = 95), posterior cingulate cortex (AD = 156, NC = 102), and temporal cortex (AD = 26, NC = 25).
- The Mount Sinai/JJ Peters VA Medical Center Brain Bank (MSBB) cohort consists of more than 2,000 well-characterized human brains and encompasses the entire range of cognitive and neuropathological disease severity, in the absence of detectable non-AD neuropathology (Wang et al., 2018). The cohort follows rigorous inclusion and exclusion criteria. Neuropathological evaluations for each sample were carried out in accordance with the Consortium to Establish a Registry for Alzheimer's Disease (CERAD) protocol. The post-mortem tissue extraction, diagnostic, and neuropsychological procedures used in this cohort were approved by the Institutional Review Boards of both Mount Sinai and JJ Peters VA Medical Center. Among RNA-seq data from MSBB's post-mortem donors, tissues such as the frontal pole (AD = 132, NC = 90), inferior frontal gyrus (AD = 132, NC = 91), parahippocampal gyrus (AD = 146, NC = 83), and superior temporal gyrus (AD = 149, NC = 86) were included. Because of the low number of samples for the prefrontal cortex (AD = 8, NC = 3), this tissue was not included.
- The Mayo Clinic Study of Aging (Mayo) is a comprehensive investigation into the prevalence, incidence, and risk factors associated with mild cognitive impairment (MCI) and dementia. The study is designed as a prospective, population-based cohort study (Roberts et al., 2008), with in-person clinical evaluations conducted either at the Mayo Clinic Abigail Van Buren Alzheimer's Disease Research Clinic or at participants' residence using standardized protocols. To confirm a diagnosis of AD, the study relies on the NINCDS-ADRDA criteria. The Mayo Clinic Study of Aging incorporates RNA-seq post-mortem donor tissue data obtained from cerebellum and temporal cortex samples. This data includes samples from both individuals with AD (84 in cerebellum, 82 in temporal cortex) and healthy individuals (78 in both cerebellum and temporal cortex).

### 2.3. RNA-seq data from genotype-tissue expression database (GTEx)

In addition, we also explored RNA-seq data from GTEx (v8) for 13 different brain tissues, including the anterior cingulate cortex, cortex, cerebellum, frontal cortex, cerebellar hemisphere, hippocampus, hypothalamus, amygdala, nucleus accumbens, caudate nucleus, putamen, spinal cord, and substantia nigra (Lonsdale et al., 2013). This resulted in a total of 2,642 samples from the 17,382 samples cataloged by GTEx across 54 tissues donated by 948 individuals. Approximately 55% of the cerebral tissue donors were between 60 and 70 years old. The RNA-seq expression data from the 13 brain tissues were preprocessed by the GTEx team, which involved aligning them with the GRCh38 genome and quantifying and normalizing each tissue using the software and techniques of STAR, RNA-Seq Expectation-Maximization (RSEM), and median gene-level transcription per million (TPM), respectively. These data were used to explore how genes are expressed at baseline.

### 2.4. Co-expression network analysis and identification of hub genes in Alzheimer's disease

To avoid processes unrelated to AD, we conducted co-expression analyses individually per group NC, AD, and samples of AD plus NC (NC+AD). The co-expression analysis was performed using *CEMiTool*, which identifies co-expression modules (Russo et al., 2018). *CEMiTool* implements an unsupervised method for gene filtering based on the inverse gamma distribution and performs a tunning for parameter selection on the identification of modules, functional enrichment analysis based on the Reactome pathway database, and drawing interaction networks. Although not developed for RNA-seq data, *CEMiTool* can process read counts by allowing the inside-built variance stabilizing transformation (VST). The tool has a dependency on *WGCNA*, a standard *R* package implemented to perform gene co-expression analysis (Langfelder and Horvath, 2008). Several *WGCNA* procedures are imported for the execution of *CEMiTool*, among which include functions for hierarchical clustering into modules and a modified version of the automatic soft-thresholding power selector function. *CEMiTool* automatically identifies the best β parameter. For RNA-seq data, *CEMiTool* recommends the use of the VST function. Therefore, input data for these analyses were kept in a non-normalized discrete distribution. The output is a user-friendly report for co-expression analysis, which include a summary of gene counts, over-representation analysis of functional pathways (ORA), gene-gene co-expression networks, gene-gene interaction networks, and hub genes. Additionally, we measured module stability by comparing the results of CEMiTool under all samples and CEMiTool by bootstrapping 70% of samples at each iteration (100x). The module stability was accessed based on the number of detected modules, elements in each module, and the number of hubs.

### 2.5. Differential co-expression network analysis

Similar to co-expression experiments, we performed differential co-expression network analysis (DCGNA) with *diffcoexp* for NC, AD, and NC+AD groups. The *diffcoexp* were performed to investigate Differential Co-expressed Links (DCLs), defined as gene pairs with statistical significance concerning the difference of the correlation coefficients under two conditions, and Differential Co-expressed Genes (DCG), genes with particularly more DCLs than expected by chance (Wei et al., 2018). *diffcoexp* has a dependency on *WGCNA* and allows the identification of pairs of genes co-expressed in at least one condition, the comparison of gene-gene correlation coefficients between each condition, and the DCGNA itself. *diffcoexp* uses Fisher's Z transformation to compare the level of correlation between pairs of genes under two conditions (case *vs* control) in order to identify DCLs. The DCGs are defined through the binomial probability model, taking into account the number of links between co-expressed pairs (Jiang et al., 2016).

### 2.6. Gene Ontology for Networks

After performing DGCNA, we converted the sub-network into the STRING network format using *Cytoscape* (Szklarczyk et al., 2023). We then visualized and analyzed the DCLs and DCGs using gene ontology (GO) analysis and *EnrichmentMap* (Isserlin et al., 2014). This approach allowed us to investigate the molecular functions and biological processes associated with enriched GO terms (FDR ≤ 0.05), providing insights into AD mechanisms.

### 2.7. Gene expression-based prediction

We used eXtreme Gradient Boosting (XGBoost, Chen and Guestrin, 2016) to fit boosted tree models for predicting AD status. Specifically, we set XGBoost to use a binary logistic objective function for the binary prediction task and ten rounds of 5-fold cross-validation. DE genes (Cavalcante et al., 2022), co-expressed gene hubs and DCGs were used as features to assess the predictive performance, which was measured by the mean Area Under Curve (AUC) and error test mean. Likewise, we examined the predictive value of differential co-expressed network sub-network using the same metrics.

## 3. Results

### 3.1. Transcripts abundance in fusiform gyrus

We identified 42,000 transcripts in the fusiform gyrus and a catalog of 30,115 well-annotated transcripts by merging RNA-seq transcripts with the Hugo Gene Nomenclature Committee from European Bioinformatics Institute. The annotated transcripts set were split into protein-coding genes (*n* = 17,829), pseudogenes (*n* = 7,111), non-coding RNAs (*n* = 4,834), and others (*n* = 341). This transcript annotation was performed aiming to focus only on protein-coding genes.

After gene annotation, the gene expression matrix was submitted to a read-filtering process before co-expression network analysis and differential co-expression analysis for quality control regarding best statistical practices. The filtering process removed non-expressed transcripts (with roughly zero expression) within samples. Thus, 18,619 transcripts remained in AD samples, 16,598 transcripts remained in NC, and 19,207 transcripts remained in the pooled NC+AD group. Subsequently, a filter per sample was performed to remove samples with gene read counts below the recommended threshold of 10 million reads. A total of six samples were removed from all our analyses, five from the AD samples and one from the NC samples.

### 3.2. Specific structure of co-expressed genes and enrichment for functional pathways in fusiform gyrus

CEMiTool's unsupervised filtering method resulted in the co-expression analysis of 1,284 transcripts for AD group, 927 transcripts for NC, and 1,303 transcripts for NC+AD samples. Co-expressed gene modules, their enriched biological pathways, as well as important hub genes associated with AD were identified by gene co-expression network analysis experiments. Overall, our analysis identified three modules in AD patients, five gene modules in NC samples, and three in pooled samples (NC+AD). For the AD, we assessed module consistency by resampling strategy (100x). Our results demonstrated high stability in identifying exactly three gene modules, with an accuracy of 74%. However, in 24% of the permutations, we found a fourth module, while only 2% of the permutations showed the presence of only two modules. On average, Module 1 (M1) contained 558.2 genes (+/-60.30), Module 2 (M2) contained 438.1 genes (+/−64.13), and Module 3 (M3) contained 135.4 genes (+/−32.90).

**Figure 1:**
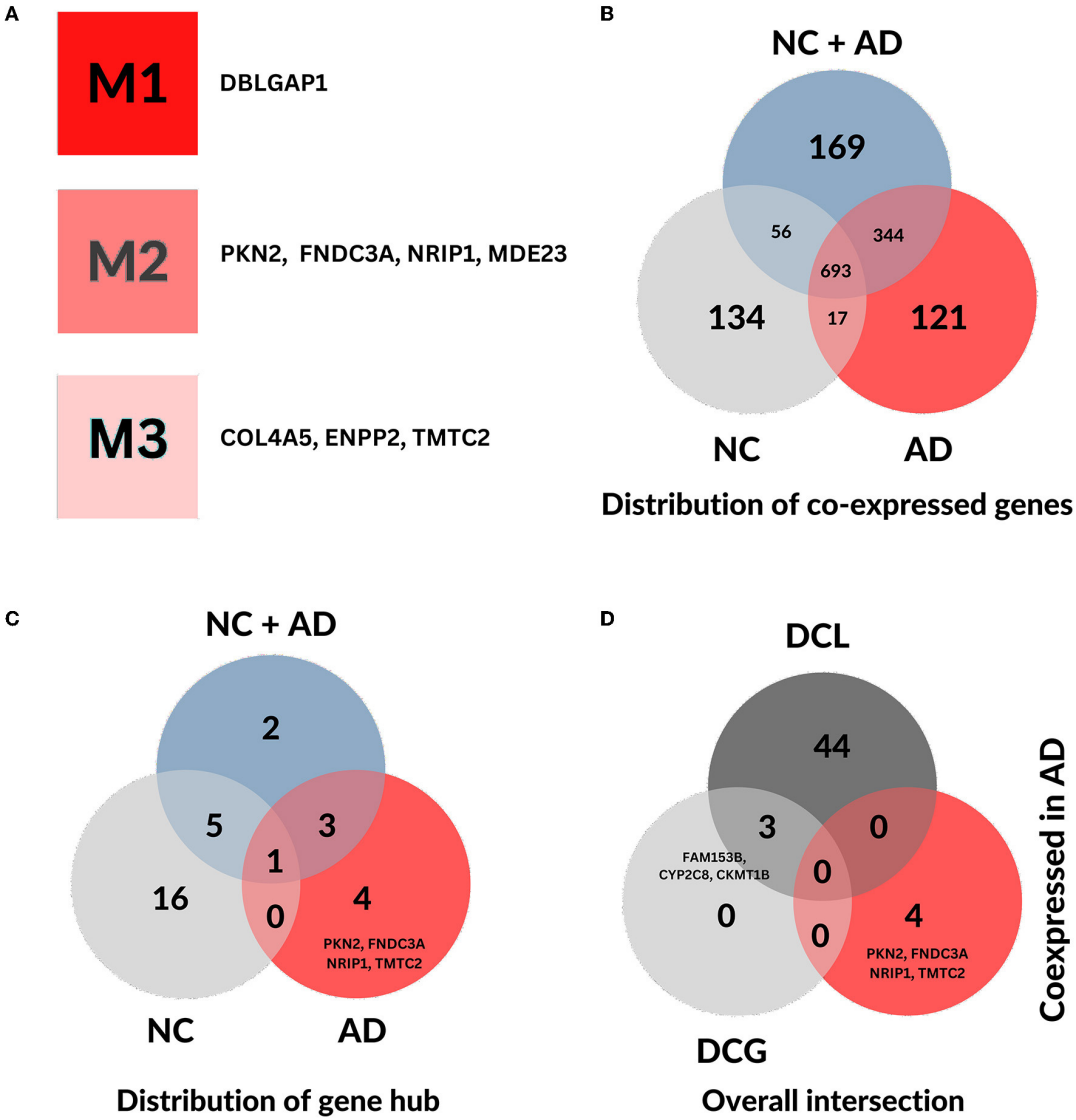
General distribution of genes from both co-expression and differential co-expression analysis. **(A)** Simplified representation of co-expressed gene hubs and their respective gene modules identified by CEMiTool in the fusiform gyrus of Alzheimer's disease. **(B)** Distribution of co-expressed genes across each condition. **(C)** Distribution of co-expressed gene hubs across each condition. **(D)** The overall intersection of co-expressed gene hubs in AD, differential co-expressed genes (DCG), and genes with differential co-expressed links (DCL) in AD.

The AD gene modules and their corresponding hubs are depicted in a clear manner in Figure 1A, while the co-expression and interaction networks of AD and NC groups are shown in Supplementary Figures S1, S2. In comparison, we note a distinction between gene modules and subsequently network structure of regulatory genes across conditions (AD and NC). With a focus on exclusive genes, 121 are AD-specific co-expressed genes (Figure 1B) and four exclusive, out of eight co-expression gene hubs, namely *PKN2, FNDC3A, NRIP1, TMTC2* (see Figure 1C). The hub genes identified in the co-expression analysis are exceptional to the analysis and do not overlap with the differential co-coexpressed network (Figure 1D), detailed in Section 3.3.

**Figure 2:**
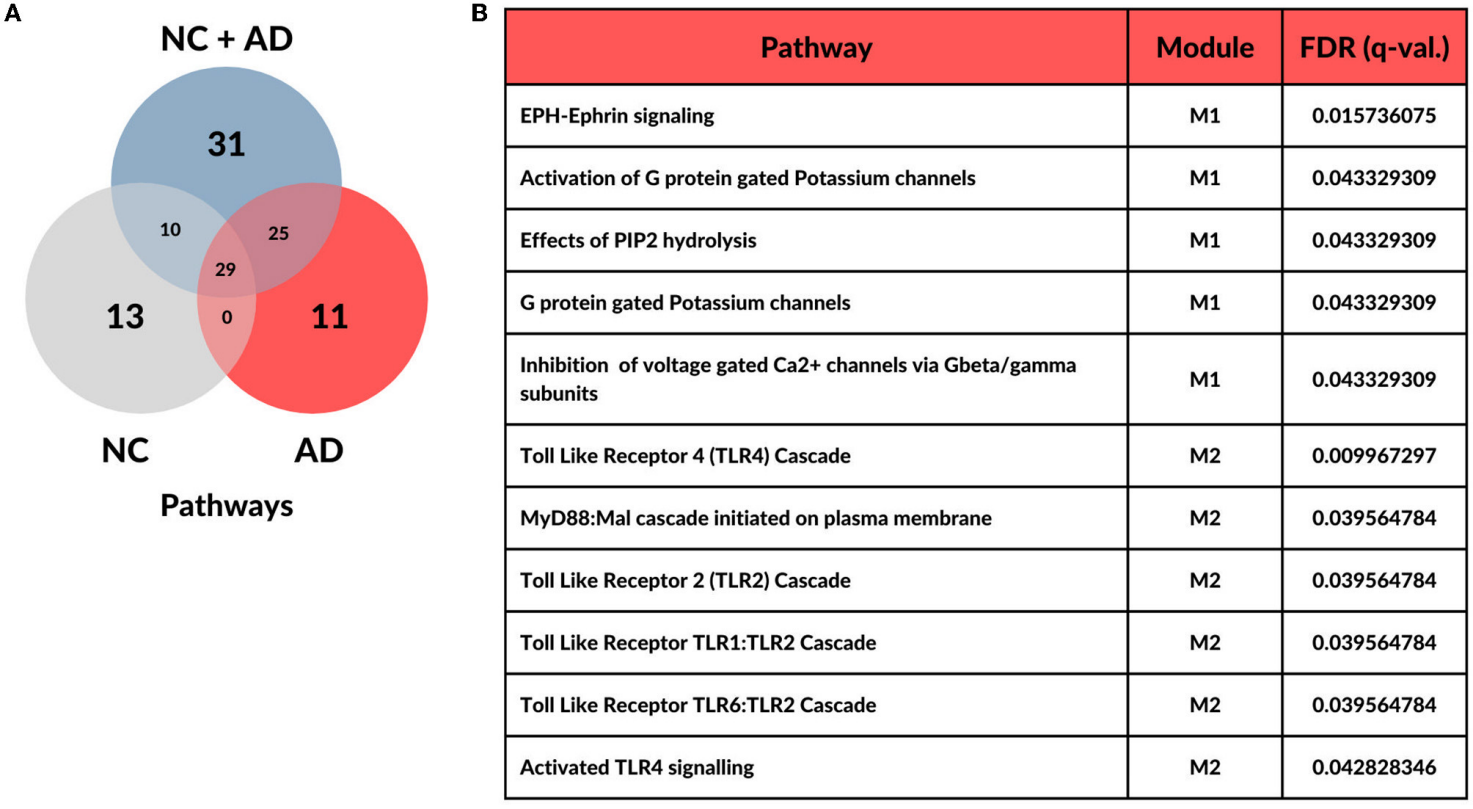
Distribution of over-represented pathways across each condition. **(A)** Overall functional enrichment analysis highlights 11 pathways with statistical significance (FDR <= 0.05) in Alzheimer's Disease. **(B)** Table with significant pathways for Alzheimer's Disease group and its respective modules.

Over-representation analysis, which was implemented in *CEMiTool*, revealed 52 functional pathways enriched for the NC group, 65 for the AD group, and 95 for the NC+AD group (adjusted p-value and q-value (FDR) ≤ 0.05). Convergences between groups are represented in the Venn diagram in Figure 2A. Among the pathways exclusive to AD, 11 were identified, comprising six Toll-like Receptors (*TLR*) cascade-related pathways and five signal transportation pathways. Notably, three of the signal transportation pathways involve membrane transport by G proteins, *PIP2* hydrolysis production of secondary messengers, and *EPH-Epherin* long-term potentiation (*LTP*) (see Figure 2B).

Interestingly, among the 75 genes described by Bellenguez et al. (2022), only seven (*TMEN106B, MS4A4A, ADAMTS1, ABCA1, HLA-DQA1, CD2AP*, and *CR1*) were not removed by CEMiTool's filtering method and underwent co-expression analysis. With the exception of *HLA-DQA1*, all genes were grouped into module 2 (M2) in AD group, which is enriched by all *TLR*-related AD-exclusive pathways. *HLA-DQA1* was not group in any module in AD samples, however, it was grouped in module 4 (M4) in NC samples.

### 3.3. Differential co-expression network and hub genes with differential co-expressed links

**Figure 3:**
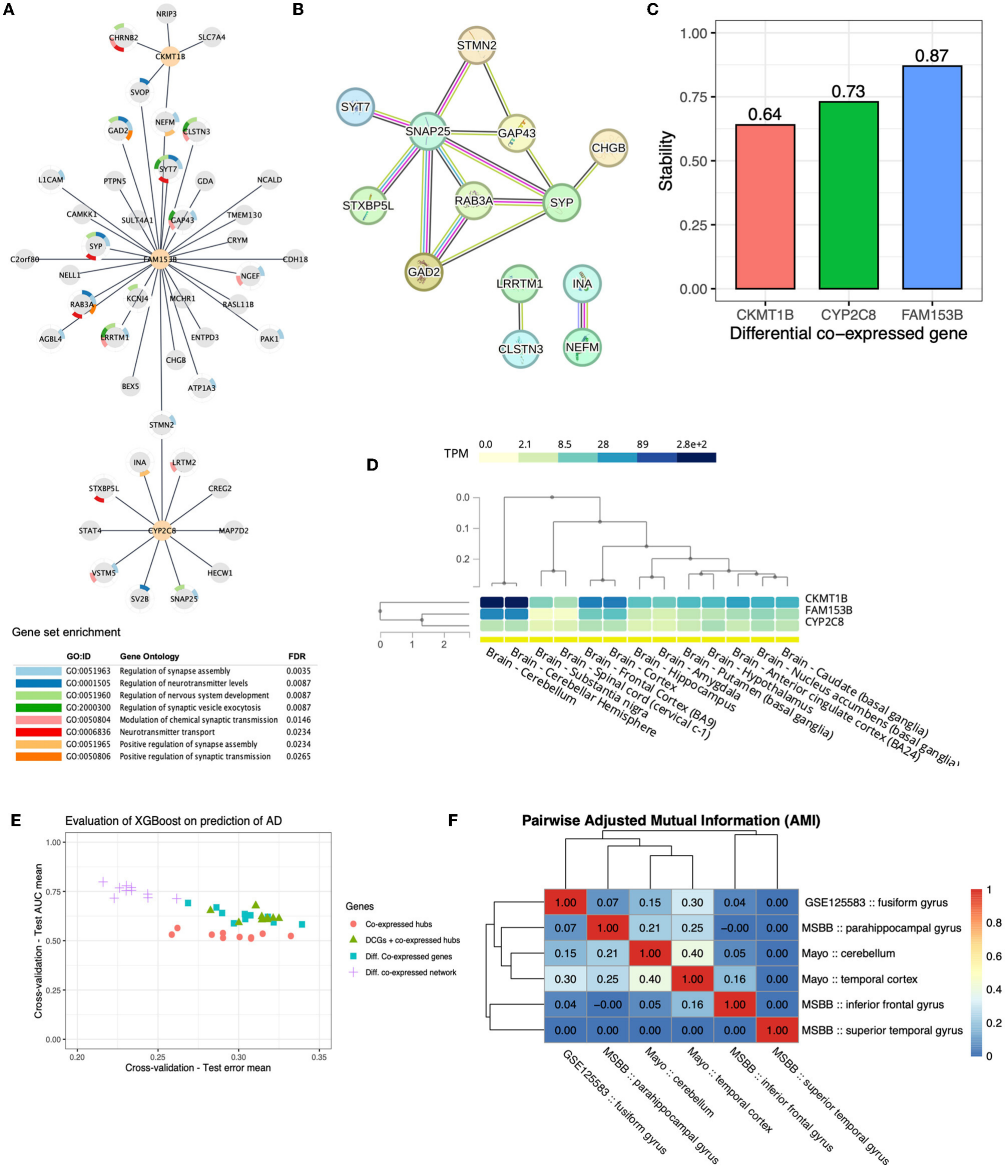
Differential co-expression Network. **(A)** Differential co-expressed sub-network identified by *diffcoexp* with 65 genes and 47 differential expressed links. The three highly connected DCGs are highlighted as orange nodes. **(B)** STRING database highlights a multi-edge protein-protein interaction sub-network for differentially expressed genes. Different colors in edges represent evidence of interaction based on co-expression. Most importantly, these interactions are evidenced by curated databases (pink edges) and experimentally determined (black edges). **(C)** Stability (accuracy) of DCGs after the 100-fold sample bootstrap tests. **(D)** Baseline expression level of the three identified DCGs on 13 tissues of normal brains cataloged in GTEx. **(E)** Test AUC mean and Test error mean of 5-fold cross-validation of XGboost regarding sets of genes. **(F)** Pairwise Adjusted Mutual Information for differential gene co-expression networks.

To perform the DGCNA, we assembled all identified genes from within the co-expressed modules found in AD or NC groups. Therefore, a total of 1,365 genes were evaluated using their links and correlations as basis. The DGCNA generated a differential co-expressed network, hereafter AD-DiffCoexpNet, that comprises 47 genes and 47 differentially co-expressed links (Figure 3, Supplementary Table 1). The AD-DiffCoexpNet overlaps with STRING interaction data, concerning 13 genes in a multi-edge protein-protein interaction network (PPI) with high confidence (>0.7) (see Figure 3B). The PPI was evidenced by curated databases, including experimental data, genomic context information, and text-mining data.

Hub analysis identified three DCGs out of a total of 47 genes in the network. The genes identified were Family with Sequence Similarity 153 Member B (*FAM153B*), which exhibited 31 DCLs, Cytochrome P450 Family 2 Subfamily C Member 8 (*CYP2C8*) with 11 DCLs, and Creatine Kinase, Mitochondrial 1B (*CKMT1B*) with five DCLs. As shown, AD-DiffCoexpNet underlined these DCGs with the highest number of DCLs compared to the other genes. For *CKMT1B, CYP2C8*, and *FAM153B*, we performed resampling analysis (100x) to assess the stability of their identification of these genes as DCGs, that accuracy values result in 0.64, 0.73, and 0.87, respectively (see Figure 3C).

Gene ontology (GO) analysis showed that the differentially co-expressed genes (DCGs) were not enriched for neuronal processes (see Figure 3A). Nonetheless, the network structure suggests that DCGs play an important role in the brain since they were found to be directly associated with genes involved in the regulation of synapse assembly, neurotransmitter levels, nervous system development, synaptic vesicle exocytosis, modulation of chemical synaptic transmission, and neurotransmitter transport (GO FDR ≤ 0.05).

Gene expression in GTEx database suggests high expression of *CKMT1B* and *FAM153B* in the cerebellar hemisphere and cerebellum (see the heatmap in Figure 3D). To get more confident molecular insights in AD, we investigated the overlap of DCGs and DCLs on AD-DiffCoexpNet in different cohorts and brain regions, including ROSMAP, MSBB, and Mayo RNA-seq data. Our analysis showed that the co-expression patterns of the AD-DiffCoexpNet are region-specific and not consistent across different cohorts and regions. Specifically, we observed a low level of adjusted mutual information between DGCNA in the fusiform gyrus and other brain regions. Figure 3F illustrates the adjusted mutual information values between the identified networks, showing a range of 0.0 to 0.4. The highest AMI is shown between the gyrus fusiform, cerebellum, and temporal cortex in the Mayo cohort, a lower similarity is observed when compared to MSBB brain regions, and no similarity is shown with the ROSMAP cohort. Despite the low AMI values (<0.40), we identified 12 genes with differential co-expression links (CDH18, BEX5, SV2B, CHRNB2, CRYM, CHGB, SVOP, GAD2, PAK1, GAP43, NELL1, RAB3A) that were common between the gyrus fusiform, the cerebellum, and the temporal cortex. These findings suggest that although the co-expression pattern (DCGs and DCLs) in the gyrus fusiform is region-specific, some gene correlations are still common across different regions. Interestingly, we did not find the three DCGs (FAM153B, CYP2C8, CKMT1B) from the fusiform gyrus in other investigated regions, which also may suggest that these DCGs are tissue-specific.

The expression-based prediction was accessed with XGBoost in a binary setting (AD x non-AD), which was trained for DE genes (Cavalcante et al., 2022), co-expressed gene hubs, DCGs, DCGs combined with co-expressed gene hubs and the entire AD-DiffCoexpNet. Of these gene sets, the expression of genes in AD-DiffCoexpNet combined with XGBoost showed moderate predictive power for diagnosing AD in the fusiform gyrus, with an average AUC test score of 0.75 (+/-0.07) and an average test error of 0.21 (+/- 0.06). Differential co-expressed gene hubs showed the lowest predictive values (AUC ≈ 0.52) as shown in Figure 3E.

## 4. Discussion

Previous studies investigated co-expression patterns in different brain tissues and identified co-expression networks for late-onset Alzheimer's disease. Zhang et al. (2013) performed a multi-tissue analysis and found strong segregation between brain regions by identifying modules using the *WGCNA* algorithm and using an approach called modular differential connectivity to find functions and pathways with significant differences across conditions. The study also identified *TYROBP* as a key regulator for the immune/microglia pathway, which was not identified in the current analysis.

Mostafavi et al. (2018) explore the frontal cortex through a system biology analysis in order to identify a molecular network to prioritize groups of genes that influence cognitive decline or neuropathology in AD. Samples were collected from two cohorts, which shared clinical and neuropathological standards, thus allowing for joint analyses. Their study used a module-trait network approach, which isolates genes into modules according to their co-expression patterns and known factors that could influence correlations, such as cell type prevalence. Modules with direct correlation to cognitive decline and other AD traits were isolated using Bayesian networks and were ranked to prioritize genes for *in vitro* validation.

Our research differed in methodological aspects. We investigate RNA-seq data from the fusiform gyrus and other large brain cohorts (ROSMAP, MSBB, and Mayo). Gene module identification was executed by *CEMiTool*, which automatizes parameter selection for the co-expression analysis, avoiding parameter-selection bias. Regarding the identification of differences across conditions, our study performed a differential co-expression analysis to focus on identifying genes/transcripts with significant differences in co-expression between groups and observing how they act in the nervous system.

Co-expression analysis and DGCNA are powerful tools that can aid in the discovery and improvement of knowledge related to the molecular architecture of complex diseases. In this study, the identification of hub genes, co-expressed gene modules, and an AD-DiffCoexpNet has provided valuable insights. Gene co-expression networks have the potential to reveal the behavior of groups of genes simultaneously, allowing for the identification of modules of correlated genes that may have potential molecular function and enrichment for functional pathways in specific conditions, such as healthy or disease cases (Chen et al., 2008). On the other hand, differential co-expression networks can identify pairs of genes with significant differences in their correlation levels between conditions, thereby highlighting regulatory elements. By utilizing these powerful tools, researchers can gain a better understanding of the molecular mechanisms underlying complex diseases. This knowledge can help to identify potential therapeutic targets and ultimately improve patient outcomes.

### 4.1. *FNDC3A, NRIP1, PKN2* and *TMTC2* are co-expressed hubs in AD modules

We identified four hub genes, namely *FNDC3A, NRIP1, PKN2*, and *TMTC2*, which were exclusively present in the AD module. These hubs were prioritized among all co-expressed hubs based on CEMiTool's gene-gene interaction networks, which indicated that they had a high degree of connectivity within their respective module's interaction networks. Furthermore, their exclusive presence in the AD co-expression modules boosts their potential relevance to AD pathology.

Previous experiments have reported Fibronectin Type III Domain-Containing 3A (*FNDC3A*) expression in the whole adult brain and odontoblasts Carrouel et al. (2008). The gene may be involved in glycosaminoglycan synthesis, which is one major part of the glycocalyx that acts on essential cell processes. Deregulated *FNDC3A* expression may impact Heparan Sulfate Glycosaminoglycan (*HSGAG*) levels, which promotes therapeutic application against AD. The knockout of *HSGAG* genes decreases the proliferation of β-amyloid fibrils in the brain (Snow et al., 2021).

Other co-expressed hubs play different roles in normal cellular function. The Nuclear Receptor-Interacting Protein 1 (*NRIP1*) plays a role in metabolic dysregulation and inflammation processes and has a dual regulation function. It negatively regulates energy homeostasis and positively regulates inflammatory response in macrophages, by indirect interaction between Nuclear Factor Kappa B (*NF-*κ*B*) and *TLR*-induced proinflammatory cytokines. Depletion of this gene was observed in the interruption of axonal degeneration (Ranea-Robles et al., 2022).

In addition, the Transmembrane O-Mannosyltransferase Targeting Cadherins 2 (*TMTC2*) encodes an integral membrane protein within the endoplasmatic reticulum (ER). The protein contains multiple clusters of tetratricopeptide domains and binds to the calcium uptake pump *SERCA2B* and to the carbohydrate-binding chaperone calnexin. Through live cell calcium measurements, Sunryd et al. (2014) report that the overexpression of *TMTC2* results in a reduction of calcium release from ER, while its knockdown stimulates calcium release, implying that the gene is involved in ER calcium homeostasis. Mutations in *TMTC2* were previously reported in sensory organ disorders, such as sensorineural hearing loss and auditory neuropathy spectrum disorder (Guillen-Ahlers et al., 2018).

#### 4.1.1. Gene co-expression analysis in fusiform gyrus reveals links with glaucoma

Based on the literature review, there is no established association between Protein Kinase N2 (*PKN2*) and neurodegeneration or direct involvement in AD development. Whereas interestingly, genetic variants, such as single nucleotide polymorphisms in exons of *PKN2* have been associated with elevated intraocular pressure, which may increase the risk for glaucoma (Gao et al., 2018). An initial study by (Wostyn et al., 2009) emphasizes links between AD and glaucoma. Interestingly, AD patients also show optic nerve degeneration and loss of retinal ganglion cells, β-amyloid and tau protein deposition in the retina Wostyn et al. (2009); Ramirez et al. (2017), and alteration of functional connectivity between visual areas dedicated to recognition like the fusiform and the inferior temporal gyri. Despite these overlaps, some links between both diseases are still under-represented, such as intraocular pressure mechanisms (Sen et al., 2020).

Associations between AD and glaucoma were also previously reported in *TMTC2* researches. Eisenhaber et al. (2021) briefly reviewed GWAS studies of the gene in ethnic-specific cohorts. The first study was performed in a Japanese cohort, claiming that *TMTC2* was a susceptible locus associated with primary open-angle glaucoma. However, follow-up studies in different ethnicity cohorts could not confirm those findings. Later, a multiethnic GWAS study identified *TMTC2* among many novel risk loci for glaucoma (Choquet et al., 2018).

Genetic comorbidity between AD and glaucoma is still unexplored, and consequently, how molecular changes affect both. Recently, Zhao et al. (2021) performed a meta-analysis of cohort studies to evaluate the association between glaucoma and AD. Based on Zhao et al. (2021), the meta-analysis concluded that glaucoma is not an independent risk factor for dementia-related diseases. Given the importance of *PKN2* in our results, we theorize that AD might play a function in the development of glaucoma disease, however, further studies are needed to support our hypothesis.

### 4.2. Over-represented pathways in AD modules

Over-representation pathway analysis in co-expression networks revealed molecular mechanisms in fusiform gyrus associated with *TLR* cascades (*TLR2, TLR4, TLR1:TLR2, TLR6:TLR2* and *MyD88:Mal* cascades), G protein signaling events (activation of potassium gates channels and inhibition of voltage-gated Ca2+ channels), *PIP2* hydrolysis, and *EPH-Epherin* mechanisms. These pathways play important roles in the immune response, synaptic transmission, and neuroplasticity, which are all processes that have been implicated in AD pathology.

Reinforcing the amyloid and tau hypothesis. We identified pathways enriched for G Proteins and consequently, for G protein-Coupled Receptors *GPCRs*, which are involved in the phosphorylation of tau through diverse downstream kinases, such as *GSK-3*β, *CDK-5* and *ERKs* signaling cascade, and interacts with β-site *APP* Cleaving Enzyme 1 (*BACE1*), both which play a major role in AD (Zhao et al., 2016; Deyts et al., 2019; Chidambaram and Chinnathambi, 2020). Huang et al. (2017) reported that *GPCRs* are successful targets for the therapeutic action on the central nervous system. Interestingly, APP/Go protein Gbeta/gamma-complex signaling was reported to mediate β-amyloid-dependent neuronal degeneration in hippocampal neurons of mice models, implying that the complex may be a promising target for therapeutic interventions in AD (Bignante et al., 2018).

Also, *TLRs* cascades trigger rapid inflammatory reactions and play a crucial role in the activation of inflammatory cascades and hypoxic-ischemic events, contributing to neuroprotective or detrimental effects of cerebrovascular diseases induced neuroinflammation (Ashayeri Ahmadabad et al., 2021; Ciesielska et al., 2021). Recently, necroptosis mediated by *TLRs* has been pointed to as a novel pathway associated with neuroinflammation (Yu et al., 2021).

The *EPH* receptors and their ligands, *Ephrins*, are involved in short-distance cell-cell signaling, regulating many neurological processes not only during development but also in adulthood. These processes include developmental cell sorting and synaptic plasticity, making the *EPH-Ephrin* signaling pathway essential for many physiological functions. Studies in AD-animal models have reported both beneficial effects and dysfunctions in synaptic plasticity and spine morphology due to *EPH* dysregulations, suggesting that the pathway might play an important role in AD (Kania and Klein, 2016). Furthermore, other studies support the association between *EPH-Epherin* signaling and AD pathogenesis. For instance, Buhl et al. (2022) investigated the effect of mutant EphA1 receptors on a Drosophila model and observed changes in behavior and neurophysiology related to AD. Meanwhile, Ganguly et al. (2022) studied the possible therapeutic implications of inhibiting the EphA-4 receptor for the targeted therapy of AD.

Phosphatidylinositol 4,5-bisphosphate (*PIP2*) hydrolysis, mediated by phospholipase C (PLC), generates two major second messengers, inositol 1,4,5-triphosphate (IP3) and diacylglycerol. Diacylglycerol activates protein kinase C (*PKC*), which plays a crucial role in the functional control of various proteins. The activation of PLCβ by Gq proteins and subsequent regulation of diverse cellular processes make them major disease drivers (Carr et al., 2021; Kankanamge et al., 2021). He et al. (2019) reports that synaptic induction of metabotropic glutamate receptor 5 (mGluR5) can hydrolyze *PIP2*, which underlies the reduced release probability in early AD (presynaptic), or can function as a β-amyloid receptor (postsynaptic). This finding suggests that an increase in presynaptic *PIP2* levels may improve cognition in AD.

### 4.3. Differential co-expressed network predicts dementia moderately

While co-expression network analysis can reveal biological mechanisms, it does not necessarily indicate causality. However, newer methods such as DGCNA can improve the identification of genes that regulate complex diseases such as Alzheimer's. We identified *FAM153B, CYP2C8*, and *CKMT1B* as highly differentially co-expressed genes with potential implications in neurodegeneration, despite not being directly enriched in neuronal-related processes. Although *FAM153B* is highly expressed in the cerebellar hemisphere and cerebellum and may have a crucial role in neurons, its function remains poorly understood, with no reported findings as of yet. In contrast, the *CYP* and *CKMT* families have been implicated in neurodegeneration. *CYP2C8* belongs to the Cytochromes P450 superfamily of enzyme-encoding genes and is involved in many metabolic pathways, that metabolize over 90% of drugs, including cholinesterase inhibitors such as tacrine, donepezil, and galantamine (Cacabelos et al., 2007).

Cholinesterase and acetylcholinesterase inhibition has shown potential in reducing neurodegenerative effects in patients with AD (Sharma, 2019), which likewise encourages the development of new cholinesterase inhibitors, since current agents may cause several side effects. While *CKMT1B* is a mitochondrial creatine kinase encoding gene commonly co-expressed with other creatine kinases and is particularly found in tissues with high-energy demands, such as the brain. The gene is considered a major target of oxidative-induced molecular damage in ischemic, cardiomyopathy, and neurodegenerative diseases (Shi et al., 2021).

Several studies have suggested that co-expressed hubs with high connectivity tend to be biologically important and have a higher likelihood of being differentially expressed, but more studies need to be performed to investigate this aspect in differential co-expressed network analysis. The relationship between the AUC of co-expression hubs and the odds of being differentially expressed or differential co-expressed is complex and may be tissue-specific. Thus, it is important to consider multiple factors and carefully evaluate the performance and biological relevance of the model in the specific context of the study. We report DCGs and DCLs in Alzheimer's disease using RNA-seq data from various brain regions and cohorts, including the GTEx database, ROSMAP, MSBB, and Mayo, that suggests our AD-DiffCoexpNet are specific to fusiform gyrus. Nonetheless, we identified 12 genes with DLCs that were common between the gyrus fusiform, cerebellum, and temporal cortex, despite low adjusted mutual information (AMI) values. We also found that some genes are still common across different brain regions, although DCGs like FAM153B, CYP2C8, and CKMT1B were not found across any other investigated regions, suggesting their tissue-specificity.

We have shown significant insights on mechanisms of Alzheimer's disease by employing state-of-the-art machine learning techniques like XGBoost, demonstrating the robustness and stability of the XGBoost models, DCGs, and AD-DiffCoepNet, despite moderate prediction (AUC = 0.75). The method incorporated DE genes, co-expressed gene hubs, DCGs, and the AD-DiffCoexpNet. Similar AUCs have been reported for genotype-based predictions (Araújo et al., 2013; Osipowicz et al., 2021). Complex diseases such as AD involve multiple genetic variants (Bellenguez et al., 2022), genes, pathways, and environmental factors, making it challenging to identify the key biomarkers and relationships that drive disease progression. The choice of machine learning algorithms and model parameters can also affect the accuracy of predictions in AD. Small datasets may lack diversity, and may not capture the full range of biological variability needed for accurate predictions, which have been improved by sharing “omics” data in public databases. It is essential to carefully evaluate and optimize each of these factors to improve the accuracy of predictions.

Differential co-expression captured the strength of association between genes in different conditions. In the case of AD-DiffCoexpNet, it is possible that the regulation of genes is influenced by transcription factors that either promote or inhibit gene expression. There could also be promoter variants present in AD samples that affect the binding of transcription factors and the subsequent interactome, ultimately leading to changes in gene expression levels. Further research is needed to confirm this hypothesis. In healthy conditions, correlated genes are often linked through shared regulatory elements, such as genetic variants, transcription factors, enhancers, or chromatin modifications. These elements can affect the expression of multiple genes simultaneously, resulting in differential co-expression patterns among related genes. This differential gene co-expression can contribute to disease progression and warrants further investigation. These findings are important for understanding the restricted specificity of gene co-expression networks in AD, which could have implications for disease progression and treatment development. While our study has provided new insights into the molecular mechanisms underlying AD and dementia pathogenesis, it is important to note that the small number of available fusiform gyrus samples is a significant limitation. Unfortunately, this has prevented us from performing experimental validation of our findings in this brain region. Therefore, further studies with larger sample sizes are necessary to confirm and extend our findings.

### 4.4. Final considerations

Our data-driven approach has led to the discovery of a valuable differentially co-expressed gene network associated with Alzheimer's disease. The AD-DiffCoexpNet is enriched with crucial neuronal-related processes, including neuron projection, synapses, and neural system development, and highlights the association of AD and complex biological gene-gene interaction networks. Our study carefully examined RNA-seq experiments from the fusiform gyrus and cross-brain regions and large brain cohorts. In fusiform gyrus, seven novel candidate genes were identified co-expressed in AD, including *FNDC3A, NRIP1, PKN2*, and *TMTC2*, as well as differentially co-expressed genes such as *FAM153B, CYP2C8*, and *CKMT1B*. In addition, the Toll-like Receptor Cascades are the most prominent pathway involved in dementia processes. To validate and strengthen our findings, we strongly encourage functional validation in longitudinal cohorts. Overall, this research significantly contributes to our understanding of the molecular mechanisms involved in Alzheimer's disease and has the potential to inform future therapeutic interventions.

## Data availability statement

The data presented in the study are deposited in the Gene Expression Omnibus (https://www.ncbi.nlm.nih.gov/geo/) repository, accession number GSE125583.

## Author contributions

All authors contributed significantly to the preparation of the manuscript, experimental study design, article writing, bioinformatics, and statistical analysis. All authors have read and agreed to the published version of the manuscript.
