# Supplementary figures and images for: The fusiform gyrus exhibits differential gene-gene co-expression in Alzheimer's disease
Source: Front Aging Neurosci. 2023 May 15;15:1138336. doi: 10.3389/fnagi.2023.1138336 (PMC10225579; doi:10.3389/fnagi.2023.1138336)

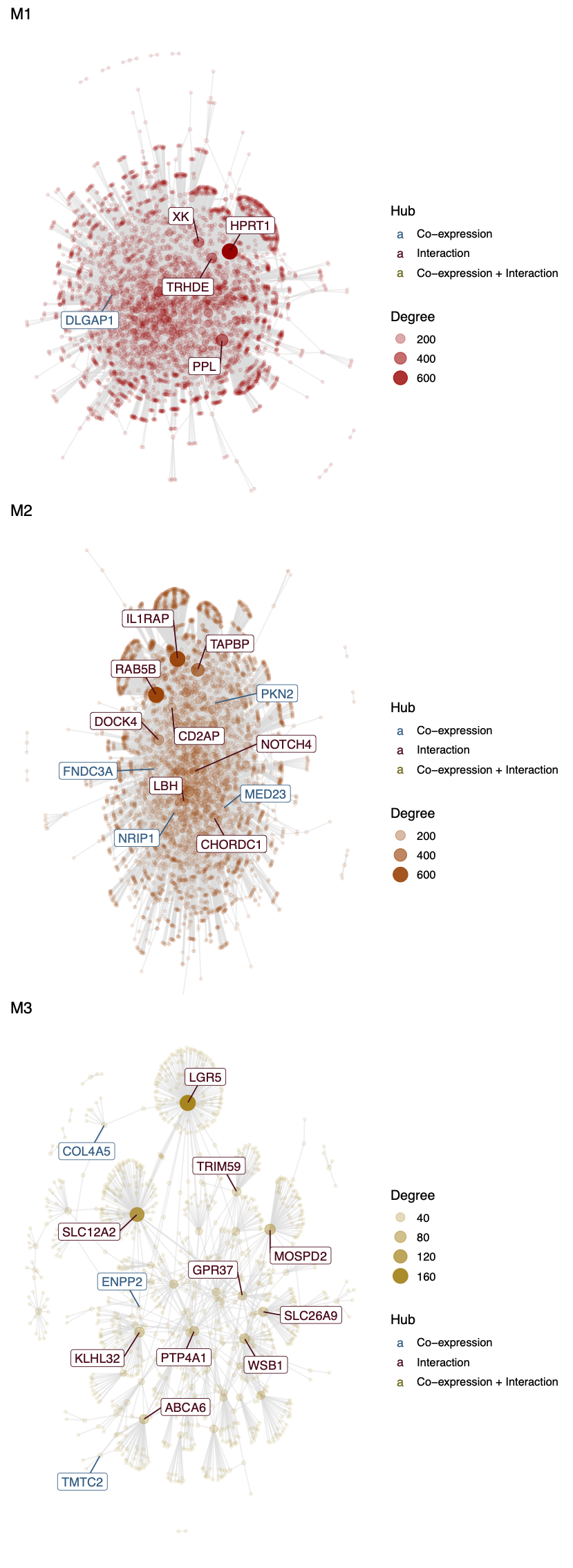

Supplement: Supplementary file 1 [file Image_1.TIFF]

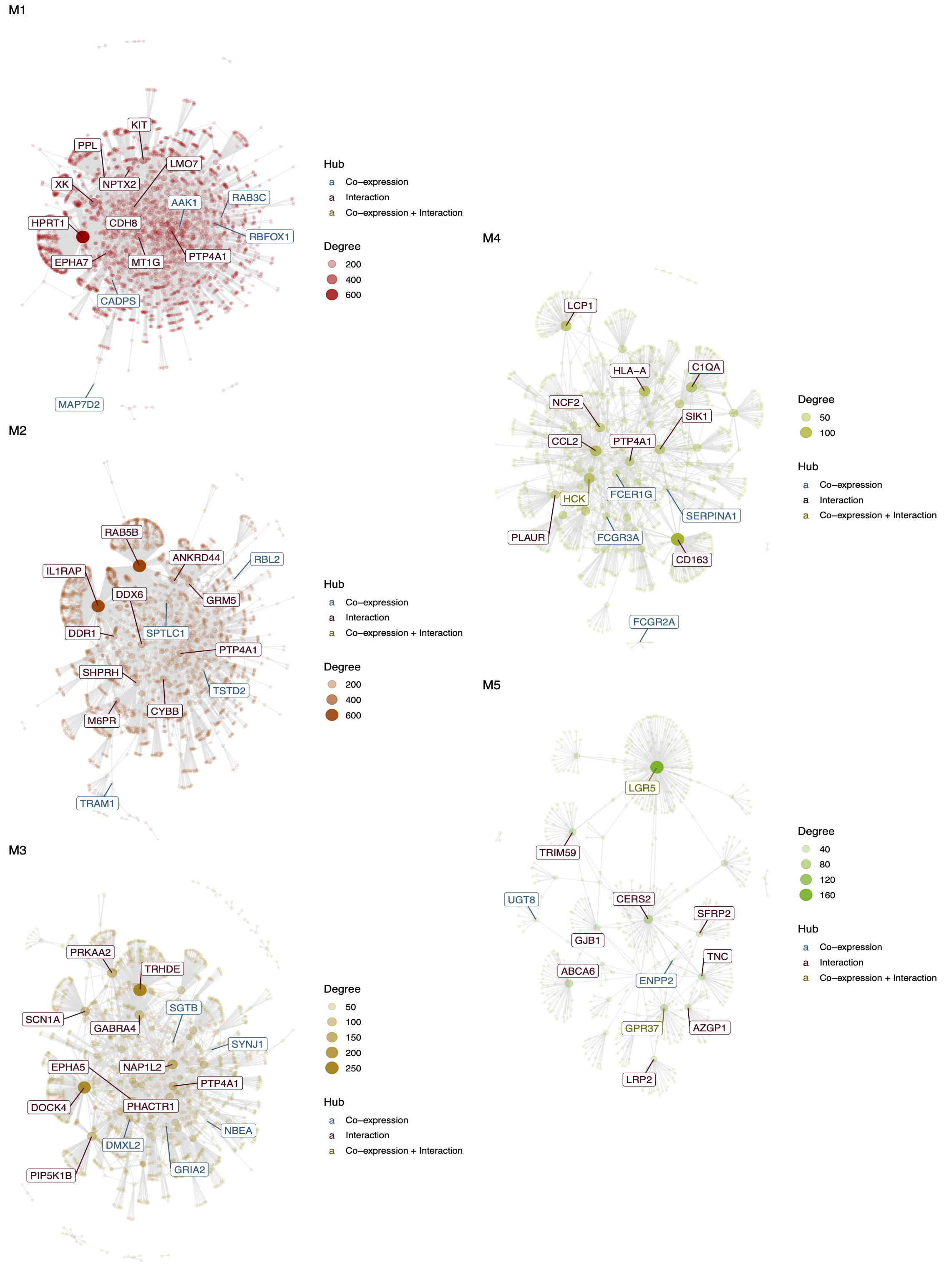

Supplement: Supplementary file 2 [file Image_2.TIFF]
