# Supplementary material for: The fusiform gyrus exhibits differential gene-gene co-expression in Alzheimer's disease
Source: Front Aging Neurosci. 2023 May 15;15:1138336. doi: 10.3389/fnagi.2023.1138336 (PMC10225579; doi:10.3389/fnagi.2023.1138336)
Supplement: Supplementary file 3 [file Data_Sheet_1.pdf]

Supplementary Material

1 SUPPLEMENTARY FIGURES

The co-expression network analysis found three gene modules in Alzheimer's disease namely M1 to M3, which show 538 519, and 118 genes, respectively. Five modules M1 to M5 were found in control samples, with 384, 208, 173, 92, and 43 genes, respectively. Modules were shown in Figures S1 and Figure 2 S2.

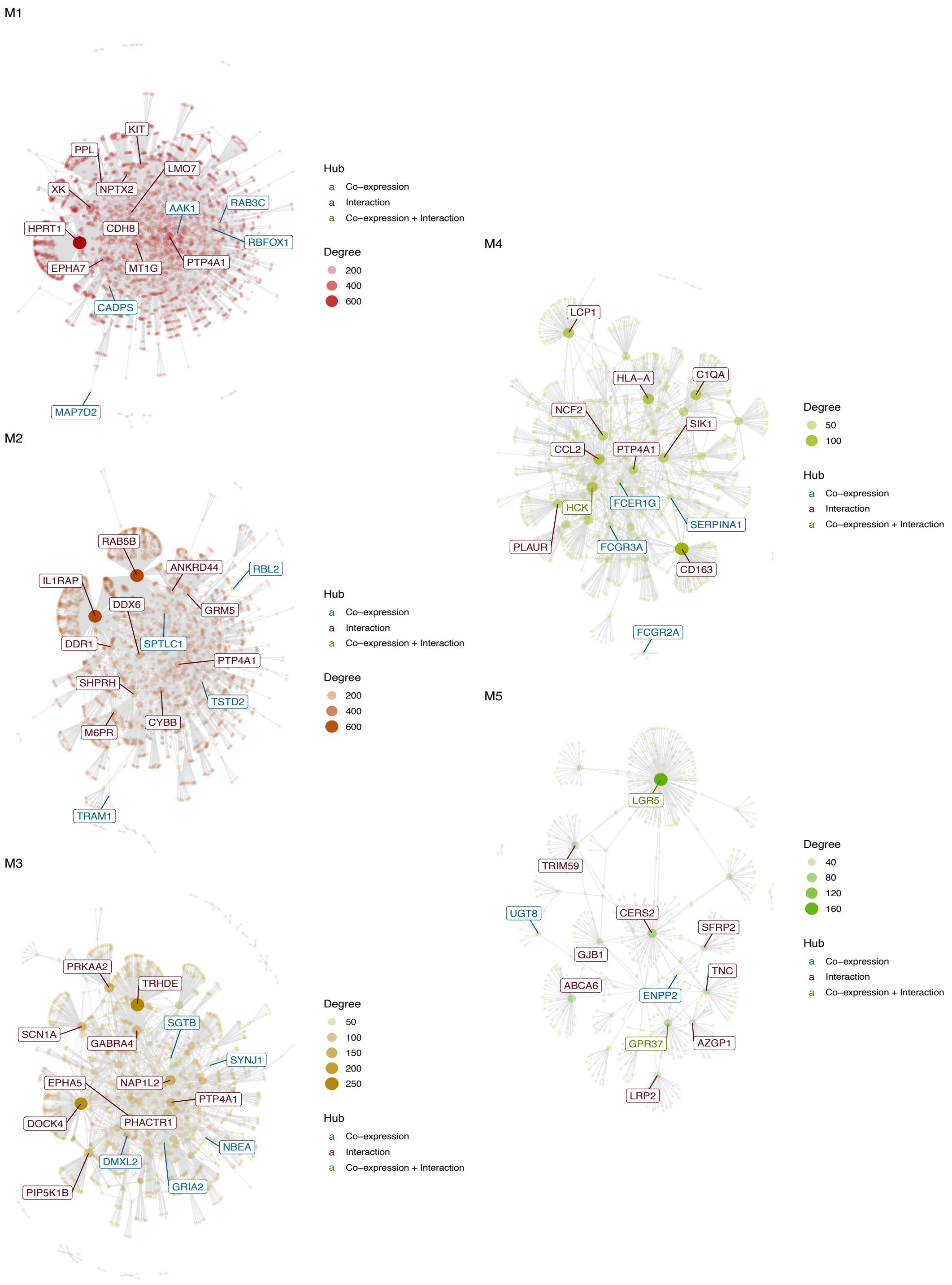

Figure S1. Gene-gene interaction networks for each co-expressed gene module identified by CEMiTool in fusiform gyrus of control samples.

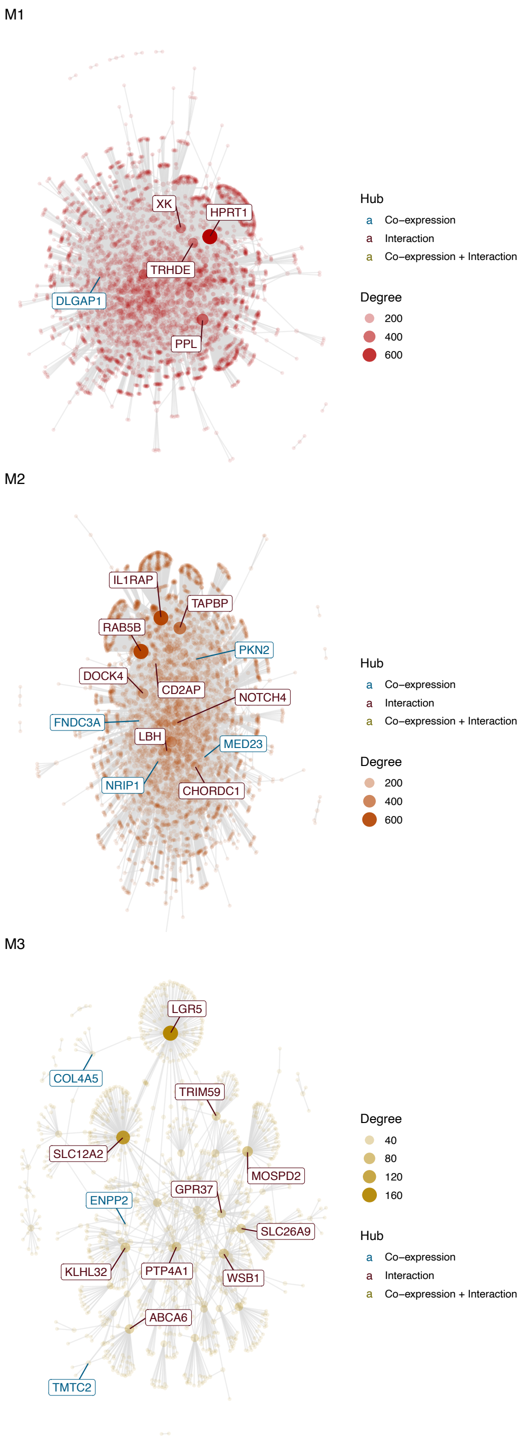

Figure S2. Gene-gene interaction networks for each co-expressed gene module identified by CEMiTool in fusiform gyrus of Alzheimer's Disease.

2 SUPPLEMENTARY TABLES

Table S1: Differentially co-expressed links identified by diffcoexp (FDR $\leq$ 0.05).

Gene A	Gene B	Corr. in Controls	Corr. in Cases	Corr. difference	FDR
**CYP2C8**	**HECW1**	0,295	0,810	0,516	1,65E-05
**FAM153B**	**CAMKK1**	0,334	0,852	0,517	2,20E-06
**FAM153B**	**SYT7**	0,376	0,895	0,518	9,69E-08
**FAM153B**	**NGEF**	0,321	0,849	0,529	1,99E-06
**FAM153B**	**CHGB**	0,242	0,807	0,565	5,46E-06
**CKMT1B**	**SLC7A4**	0,240	0,817	0,576	2,54E-06
**FAM153B**	**SYP**	0,270	0,834	0,563	1,99E-06
**FAM153B**	**CRYM**	0,351	0,869	0,518	1,17E-06
**CYP2C8**	**STMN2**	0,289	0,813	0,524	1,25E-05
**FAM153B**	**STMN2**	0,344	0,850	0,506	2,75E-06
**FAM153B**	**NCALD**	0,317	0,839	0,522	3,18E-06
**FAM153B**	**NEFM**	0,260	0,824	0,563	2,54E-06
**CKMT1B**	**NEFM**	0,309	0,816	0,507	1,65E-05
**FAM153B**	**ATP1A3**	0,266	0,812	0,546	7,11E-06
**FAM153B**	**RAB3A**	0,298	0,834	0,535	3,18E-06
**FAM153B**	**PTPN5**	0,366	0,879	0,512	8,35E-07
**FAM153B**	**GDA**	0,365	0,869	0,504	1,67E-06
**FAM153B**	**RASL11B**	0,322	0,854	0,532	1,73E-06
**FAM153B**	**MCHR1**	0,223	0,811	0,588	2,54E-06
**FAM153B**	**SULT4A1**	0,329	0,856	0,527	1,73E-06
**CYP2C8**	**SNAP25**	0,307	0,815	0,507	1,71E-05
**FAM153B**	**GAD2**	0,301	0,807	0,506	2,37E-05
**STAT4**	**CYP2C8**	0,277	0,819	0,542	6,02E-06
**STXBP5L**	**CYP2C8**	0,231	0,805	0,573	5,02E-06
**INA**	**CYP2C8**	0,307	0,823	0,517	9,57E-06
**LRTM2**	**CYP2C8**	0,300	0,806	0,505	2,44E-05
**CREG2**	**CYP2C8**	0,301	0,804	0,503	2,82E-05
**MAP7D2**	**CYP2C8**	0,296	0,814	0,519	1,35E-05

Table S1 continued from previous page

Gene A	Gene B	Corr. in Controls	Corr. in Cases	Corr. difference	FDR
**SV2B**	**CYP2C8**	0,302	0,805	0,503	2,64E-05
**VSTM5**	**CYP2C8**	0,251	0,816	0,565	3,40E-06
**FAM153B**	**CLSTN3**	0,322	0,846	0,523	2,42E-06
**FAM153B**	**CDH18**	0,315	0,845	0,530	2,27E-06
**FAM153B**	**PAK1**	0,324	0,854	0,530	1,73E-06
**CKMT1B**	**CHRNB2**	0,343	0,853	0,510	2,36E-06
**FAM153B**	**LRRTM1**	0,305	0,838	0,533	2,60E-06
**FAM153B**	**NELL1**	0,314	0,827	0,513	8,51E-06
**FAM153B**	**SVOP**	0,248	0,824	0,575	2,20E-06
**CKMT1B**	**SVOP**	0,321	0,853	0,532	1,73E-06
**FAM153B**	**TMEM130**	0,307	0,850	0,543	1,73E-06
**FAM153B**	**ENTPD3**	0,317	0,846	0,529	2,27E-06
**FAM153B**	**KCNJ4**	0,298	0,822	0,524	8,11E-06
**FAM153B**	**GAP43**	0,259	0,832	0,573	1,73E-06
**CKMT1B**	**NRIP3**	0,305	0,813	0,509	1,77E-05
**BEX5**	**FAM153B**	0,263	0,825	0,561	2,54E-06
**AGBL4**	**FAM153B**	0,279	0,803	0,524	1,80E-05
**C2orf80**	**FAM153B**	0,330	0,869	0,539	8,35E-07
**L1CAM**	**FAM153B**	0,330	0,830	0,500	1,02E-05
